# Effects of down-regulation of *ackA* expression by CRISPR-dCpf1 on succinic acid production in *Actinobacillus succinogenes*

**DOI:** 10.1186/s13568-023-01518-x

**Published:** 2023-01-26

**Authors:** Chunmei Chen, Pu Zheng

**Affiliations:** The Key Laboratory of Industrial Biotechnology, Ministry of Education, School of Biotechnology, Jiangnan University, Wuxi, 214122 China

**Keywords:** CRISPRi, *Actinobacillus succinogenes*, Succinic acid, Acetic acid, *ackA*

## Abstract

Succinic acid (SA), a key intermediate in the cellular tricarboxylic acid cycle (TCA), is a 4-carbon dicarboxylic acid of great industrial value. *Actinobacillus succinogenes* can ferment various carbon sources and accumulate relatively high concentrations of SA, but few reliable genetic engineering tools exist for *A. succinogenes* and this has hindered strain improvement to increase SA production for industrial application. Two different repressors, endonuclease-deactivated Cas9 (dCas9) from *Streptococcus pyogenes* and Cpf1 (dCpf1) from *Francisella tularensis*, were applied to construct a CRISPRi system in *A. succinogenes*. Codon-optimized Cas9 and native Cpf1 were successfully expressed in *A. succinogenes*, and the corresponding sgRNA and crRNA expression elements, promoted by the fumarate reductase promoter, *frd*, were introduced into the CRISPRi plasmid. The highest repression of the *ackA* gene (encoding acetate kinase) and thereby acetic acid production (~ eightfold) was achieved by the dCpf1-based CRISPRi system, in which the mutation site, E1006A acted at the start of the coding region of *ackA,* the gene which regulates acetic acid biosynthesis. Compared with the *ackA* gene knockout mutant, cell growth was moderately improved and SA production increased by 6.3%. Further, the SA titer and productivity in a 3 L fermenter reached 57.06 g/L and 1.87 g/L/h, and there was less acetic acid production. A dCpf1-based CRISPRi-mediated gene repression system was successfully established for the first time, providing a simple and effective tool for studying functional genomics in *A. succinogenes* and optimizing SA production.

**Supplementary Information:**

The online version contains supplementary material available at 10.1186/s13568-023-01518-x.

## Keypoints

- CRISPRi based on FnCpf1 and SpCas9opt was established in *A. succinogenes*.
- The target gene, ackA was repressed ~ eightfold by CRISPR-dCpf1.
- Repression of ackA increased succinate yield compared with ackA deletion.

**Supplementary Information:**

The online version contains supplementary material available at 10.1186/s13568-023-01518-x.

## Introduction

Production of materials and chemicals from renewable resources by fermentation with microbial cell factories is a promising and more sustainable alternative to conventional chemical synthesis (Tong et al. 2020; Mika et al. 2018; Khoshnevisan et al. 2020). Succinic acid (SA), a 4C-dicarboxylic acid, is a key intermediate in the cellular TCA cycle and has numerous applications in agriculture, green solvents, pharmaceuticals, and biodegradable plastics (Liu et al. 2020). The United States Department of Energy (DOE) classifies SA as one of the 12 value-added bio-based “platform chemicals” (Su et al. 2021). Recently, SA has been recognized as an important precursor for production of biological base chemicals, such as biodegradable plastics (e.g. polybutylene succinate), polyester polyols, plasticizers, polyurethanes and 1,4-butanediol (Kumar et al. 2020).

Many microorganisms, such as *Actinobacillus succinogenes*, *Escherichia coli*, *Corynebacterium glutamicum*, *Mannheimia succiniciproducens*, *Zymomonas mobilis*, *Saccharomyces cerevisiae*, *Aspergillus nige*r, and *Yarrowia lipolytica* can be used for sustainable fermentative production of SA, using various renewable bioresources (Yu et al. 2019; Huang et al. 2019; Widiastuti et al. 2018; Chong et al. 2016; Franco-Duarte et al. 2017; Chen et al. 2019; Ahn et al. 2020; Putri et al. 2020; Gonzales et al. 2020; Yang et al. 2020a, b). *A. succinogenes*, a Gram-negative, capnophilic and facultative anaerobic bacterium, naturally produced a high yield of SA from a variety of carbon sources, was initially isolated from bovine rumen, and was one of the most promising strains for bioproduction of SA (Putri et al. 2020; Yang et al. 2020a, b; Dessie et al. 2018; Guettler et al. 1999). Although *A. succinogenes* produces a high SA yield, a lack of reliable genetic engineering tools has hampered strain improvement to overcome several limiting factors in SA production. The first expression vector pLGZ920 and its derivatives were successfully constructed, based on the shuttle plasmid pGZRS-19, which can recombinantly express exogenous proteins in *A. succinogenes* (Kim et al. 2004). Using plasmid pLGZ922, an exogenous glutamate decarboxylase (Gad) system was introduced into *A. succinogenes* CGMCC 1953, which improved acid resistance (Chen et al. 2022). A knockout strategy using markerless knockout was developed, based on the auxotrophy of *A. succinogenes* for glutamate and selection for growth on isocitrate, using the *E. coli* isocitrate dehydrogenase gene as a positive selection marker and natural transformation or electroporation. In addition, the *Saccharomyces cerevisiae* flippase/recombinase (Flp) system was used to remove the positive selection marker and the genes encoding fumarate reductase and pyruvate formate lyase were deleted. Deletion mutants of citrate lyase, galactosidase, and aconitase were made from the pyruvate formate lyase knockout mutant (Joshi et al. 2014). Subsequently, simpler knockout protocols using conventional antibiotic resistance markers were reported. Homologous recombination-mediated chromosomal integration and gene disruption were achieved through genomic homology regions flanking an antibiotic resistance marker. This method was used for SA biosynthetic gene overexpression and competitive carbon pathway knockout (Guarnieri et al. 2017). In addition, an alternative method for markerless gene deletion by allelic exchange in *A. succinogenes* was also developed; the pyruvate formate lyase 1-activating protein encoded by *pflA* in *A. succinogenes* 130Z was successfully knocked out by in-frame deletion, to generate the mutant (Zhang et al. 2019a, b). However, the above-mentioned processes are relatively complicated, time-consuming and inefficient, the SA titer of engineering strains was not increased significantly.

In recent years, the Clustered Regularly Interspaced Short Palindromic Repeats (CRISPR)/Cas genome editing system has been developed; its simplicity and operability have facilitated its use in many microorganisms, such as *E. coli*, *C. glutamicum*, *Candida tropicalis* and *Bacillus megaterium* (Li et al. 2021; Liu et al. 2017; Zhang et al. 2019a, b; Hartz et al. 2021). Most of these CRISPR genome editing systems were adapted from the Type II Cas9 and Cpf1 endonucleases from the bacterial antiviral defense system (Shi et al. 2021; Du et al. 2021; Liu et al. 2022). dCas protein, which has no endonuclease activity, was generated by mutating key amino acid residues in the endonuclease domain of the Cas protein. CRISPR interference (CRISPRi) was developed to repress gene expression by dCas protein, by binding to the promoter, or coding region of the target gene and blocking the access of RNA polymerase (RNAP) (Wu et al. 2018; Li et al. 2020; Banta et al. 2020). To the best of our knowledge, no CRISPR/Cas system has been reported in *A. succinogenes*.

In previous studies, high SA production by *A. succinogenes* was found to be accompanied by high acetic acid (AA) production. To obtain a high yield of SA, it is necessary to maximize metabolic flux towards SA and minimize flux to alternative products, such as AA, through metabolic engineering; inhibiting AA production by *A. succinogenes* is an important research goal (Yang et al. 2020a, b). Dynamic regulation of AA synthesis by a switchable tool is a promising way to balance cell growth and SA biosynthesis. In this study, the pLGZ922 vector was used to express Cas protein, controlled by its promoter *pckA*, then a fumarate reductase promoter was used to generate a gRNA scaffold. Finally, a CRISPRi-mediated gene repression system, based on dCpf1, was established in *A. succinogenes*, which can efficiently repress specific target genes. A higher titer of SA and higher cell growth were achieved by down-regulation of the *ackA* gene in *A. succinogenes* by CRISPRi, than from an *ackA* knockout mutant.

## Materials and methods

### Strains and media

*Escherichia coli* JM109 was used for gene cloning. *A. succinogenes* CGMCC1593 was isolated from bovine rumen in our laboratory and stored at the China General Microbiological Culture Collection Center. Luria–Bertani (LB) medium (10 g/L tryptone, 5 g/L yeast extract, and 10 g/L NaCl) was used for *E. coli* culture. Tryptic soy broth (TSB) medium (Sinopharm Chemical Reagent Co., Ltd, China) was used for *A. succinogenes* culture (Chen et al. 2022).

### Construction of *A. succinogenes ackA* gene knockout strain

The primers, strains and plasmids used are listed in Additional file 1: Tables S1 and S2. To delete the *ackA* gene from *A. succinogenes*, a knockout cassette was constructed containing a 1.0 kb up-stream region, a kanamycin label and a 1.0 kb downstream region of the *ackA* gene. The homologous arms were amplified from the genome of *A. succinogenes* and the kanamycin label was amplified from plasmid pET28a by PCR using the primers in Additional file 1: Table S1. The three fragments were connected by fusion PCR using PrimeSTAR Max Premix (TaKaRa, BeiJing, China), then cloned into plasmid pCVD442, to produce the target plasmid pCVD442-ackA. The target plasmid was naturally transformed into *E. coli* DH5α λpir. Positive colonies were screened on LB plates containing 25 mg/mL kanamycin and 50 mg/mL ampicillin. The plasmid was extracted from a positive colony and used to transform the donor strain *E. coli* β2155 using electroporation (voltage 1700 kV). Positive colonies were screened on LB plates containing 25 mg/mL kanamycin and 0.5 mM diaminopimelic acid (DAP).

Recipient *A. succinogenes* was cultured anaerobically in TSB medium overnight at 37 °C, and donor strain *E. coli* β2155 in LB medium overnight at 37 °C. Donor and recipient cell suspension (0.5 mL each) were placed into two sterilized 1.5 mL tubes. The cells were harvested by centrifugation (3000 ×*g*, 5 min) and resuspended with TSB medium. The cells were harvested again by centrifugation (3000 ×*g*, 5 min) and mixed in one tube using TSB medium containing DAP (0.1 mM). Mixed cell suspension (100 μL) was added to conjugation plates containing DAP (0.1 mM), then cultured anaerobically at 30 °C for 6 h. The cells were resuspended in TSB medium (1 mL) and spread onto TSB plates containing 25 mg/mL kanamycin, then cultured anaerobically at 30 °C until colonies formed. The clones from the first cross-over were screened by PCR and the positive clones were cultured anaerobically overnight at 30 °C. The cells were harvested by centrifugation (3000 ×*g*, 5 min) and resuspended with LB medium containing 10% sucrose and no NaCl. The cells were harvested again by centrifugation (3000 ×*g*, 5 min) and washed three times with LB medium containing 10% sucrose and no NaCl. The cells was resuspended again with LB medium (2 mL, 0 g NaCl, 10% sucrose) and incubated anaerobically at 30 °C overnight. Finally, the cultures were spread onto TSB plates containing 25 mg/mL kanamycin and incubated at 30 °C anaerobically until colony formation. The mutants were screened by PCR and sequencing.

### Western blotting

*Actinobacillus succinogenes* containing Cas protein was cultured anaerobically in TSB medium at 37 °C for 24 h, then the cells were harvested by centrifugation at 6000 ×*g* for 10 min and resuspended in sterile PBS. The cells were ultrasonicated (3 s on/5 s off, for 20 min at 350 W), then the suspension was centrifuged at 6000 ×*g* for 10 min. The supernatant, a crude protein preparation, was subjected to SDS-PAGE, with a 6% separation gel and a 5% stacking gel.

After SDS-PAGE, the gel was subjected to wet membrane translocation, then TSBT (Tris 1.21 g/L, NaCl 8.77 g/L, pH 7.5) solution was used to remove the membrane transfer solution, the membrane was soaked in 5% milk powder solution for 1 h, then soaked in TSBT solution for 10 min, repeated three times. Anti-6 × His Tag mouse monoclonal antibody (WB kit, Sangon Biotech) was added and incubated overnight on ice, membrane washed in TSBT solution three times, then the secondary antibody was added and incubated for 1 h. Finally, the membrane was washed in TSBT solution three times and HCL solution was added to observe the protein bands.

### CRISPRi plasmid construction

The plasmid pLGZ922, containing a *pckA* promoter, an *A. succinogenes* replication origin and an ampicillin resistance label was employed for Cas gene expression. The *frd* promoter was amplified from the *A. succinogenes* genome. The codon-optimized *cas9* gene (*cas9opt*) was synthesized by GENEWIZ (Suzhou, China), then the *kcas9* gene was amplified from plasmid pKCcas9dO, and the *cpf1* gene was amplified from plasmid pDZLcas12a (Huang et al. 2015; Zhou et al. 2020). Several *cas* genes were amplified by PCR and cloned into plasmid pLGZ922 with a one-step cloning kit (Vazyme, Nanjing, China), generating the Cas expression plasmids (Additional file 1: Table S2). The Cas expression plasmids were subjected to site-directed mutation by the relevant primers to generate new plasmids containing devitalized Cas proteins.

The *frd* promoter was amplified from *A. succinogenes* and the sgRNA scaffold was amplified from plasmid pKCcas9dO by PCR using the relevant primers (Additional file 1: Table S2), then the *frd* promoter and sgRNA scaffold were connected by fusion PCR. Finally, the fusion fragments were cloned into the expression plasmid of the *cas9* gene using a one-step cloning kit (Vazyme), generating CRISPRi-dcas9 plasmids.

Similarly, the *frd* promoter was amplified from *A. succinogenes* and the crRNA scaffold was amplified from plasmid pUCLcrRNA by PCR, using the relevant primers. The *frd* promoter and crRNA scaffold were connected by fusion PCR. Finally, the fusion fragments were cloned into the expression plasmid of the *cpf1* gene by the one-step cloning kit (Vazyme), generating CRISPRi-dCpf1 plasmids.

### Fermentation

The *A. succinogenes* strains were cultured in 50 mL shake flasks containing 25 mL TSB medium overnight at 37 °C in an anaerobic incubator, then a 2.5% v/v inoculum was added to a second seed medium at 37 °C for 10–14 h (pH ~ 6.). A 10% suspension was inoculated into fermentation medium with suitable Mg(CO_3_)_2_ and the pH was maintained at 6.0–6.5 with 300 g/L Na_2_CO_3_. The temperature and agitation were 38 °C and 200 rpm, respectively. The glucose content was maintained between 10 and 20 g/L by adding glucose; the glucose concentration was monitored by HPLC as described below.

### Analytical methods

The optical density of *A. succinogenes* was monitored by spectrophotometry at 660 nm (OD_660_). Glucose and organic acids in fermentation broth were analyzed by HPLC with a Waters system fitted with a Sepax Carbomix H-NP column (Sepax Technologies, Newark, DE) and a refractive index (RI) detector. The column temperature was 55 °C and the mobile phase was 3.3 mM H_2_SO_4_ at a flow rate 0.5 mL/min.

## Results

### Knockout of *ackA* gene by homologous recombination

**Fig. 1:**
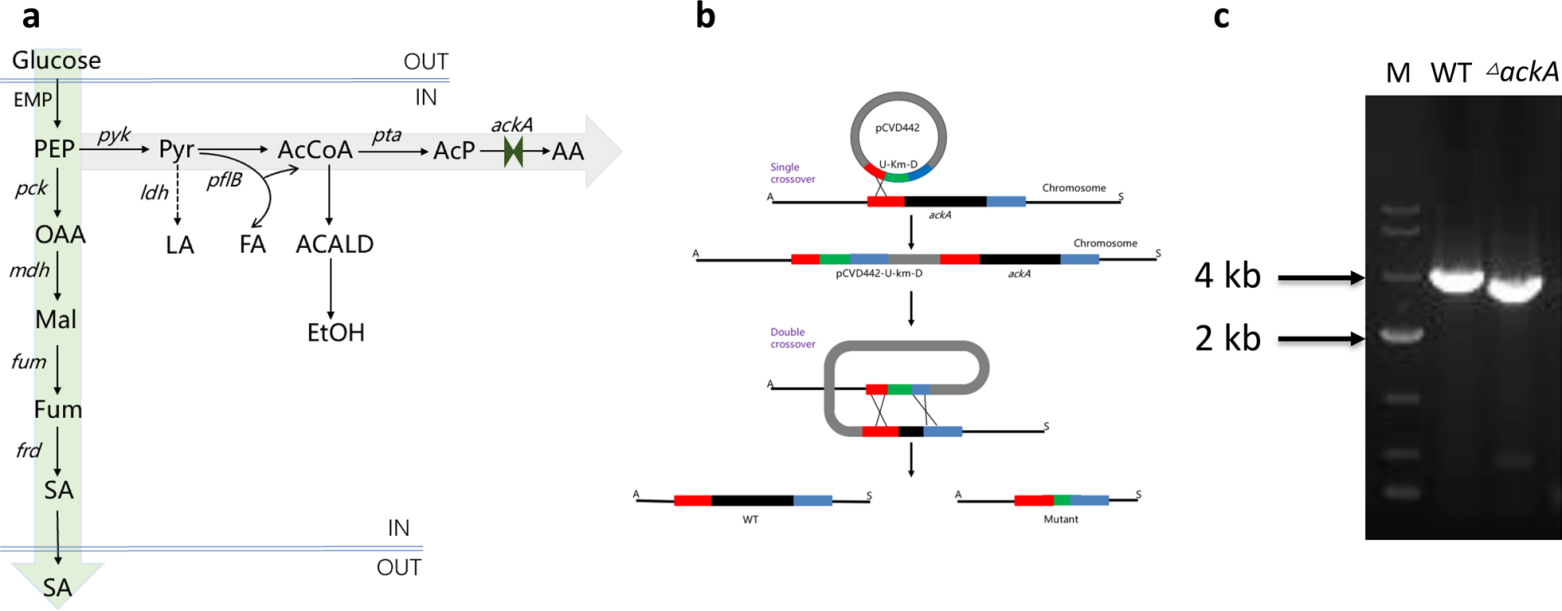
The central metabolic network for SA biosynthesis in *A. succinogenes* (**a**) (PEP, Phosphoenolpyruvate; Pyr, Pyruvate; LA, lactic acid; AA, acetic acid; FA, formic acid; ACALD, acetaldehyde; EtOH, Ethanol; OAA, oxaloacetate; Mal, malate; Fum, fumarate; SA, succinic acid; AcCoA, acetyl-CoA; AcP, acetyl-phosphate; *pck*, phosphoenolpyruvate carboxykinase; *mdh*, malate dehydrogenase; *fum*, fumarase; *frd*, fumarate reductase; *pyk*, pyruvate kinase; *ldh*, lactate dehydrogenase; *pflB*, pyruvate formate-lyase; *pta*, phosphoacetyltransferase); Schematic diagram of homologous recombination for *ackA* gene knockout (**b**); PCR analysis of the candidate *A. succinogenes* colonies **(c)** (M, 10 kb DNA ladder; WT, 3390 bp; Δ*ackA*, 3114 bp)

Succinic acid (SA) is mainly produced by *A. succinogenes* from glucose and CO_2_ by the C4 pathway, however, some PEP flows through the C3 pathway during fermentation, producing acetic acid (AA) (Fig. 1a) (Hijosa-Valsero et al. 2022; Shen et al. 2022). To maximize the SA yield, one approach is to knockout the *ackA* gene, coding acetate kinase, which is responsible for acetic acid production by the C3 pathway. The plasmid pCVD442 was employed, with ampicillin resistance and *sacB* genes. The knockout plasmid contained a fusion fragment with the 1.0 kb upstream region, kanamycin label and 1.0 kb downstream region of the *ackA* gene. The knockout mutant containing kanamycin resistance was obtained by single cross-over and double cross-over screening (Fig. 1b). The mutant was identified by colony PCR and sequencing (Fig. 1c).

### CRISPR/Cas protein screening

**Fig. 2:**
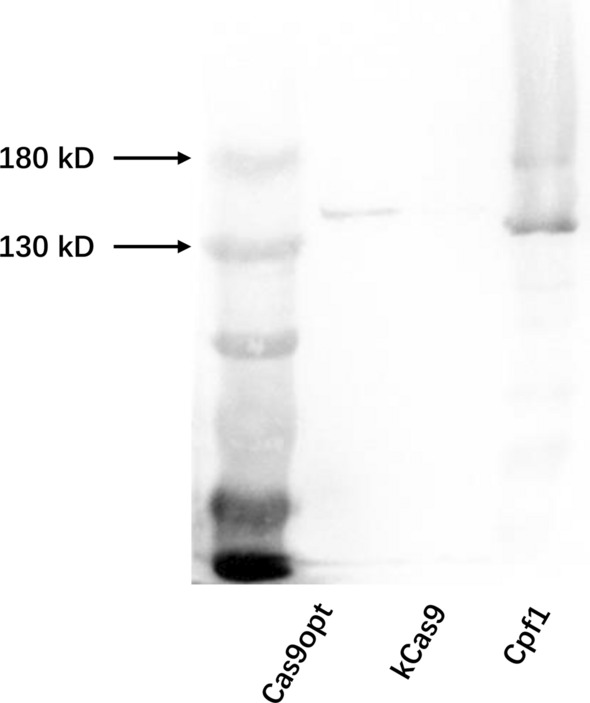
Western blot analysis of Cas proteins (M, 180 kD protein ladder; Cas9opt, cas9 codon-optimized for *A. succinogenes*; kCas9 from plasmid pKCcas9dO; Cpf1 from plasmid pDZLcas12a)

Most of the current CRISPR tools are modified from type II CRISPR/Cas9 and CRISPR/Cpf1. To establish a CRISPR/Cas system in *A. succinogenes*, several Cas proteins were screened, including Cas9 from *Streptococcus pyogenes* from plasmid pKCcas9dO, the codon optimized Cas9 from *S. pyogenes* and Cpf1 from *Francisella tularensis* from plasmid pDZLcas12a (Huang et al. 2015; Zhou et al. 2020) The expression plasmid pLGZ922 was applied to express Cas protein, and a 6 × His tag was added to the C-terminus of Cas proteins. A strong promoter, *pckA* was used to maximize Cas protein expression and the effectiveness of Cas protein expression in *A. succinogenes* was determined by Western blotting (Fig. 2); Cas9opt and Cpf1 were expressed effectively, but not kcas9. This indicated that Cas9opt and Cpf1 could be employed as effective Cas proteins for gene editing in *A. succinogenes*.

### CRISPRi mediated gene repression base on Cas9 in *A. succinogenes*

**Fig. 3:**
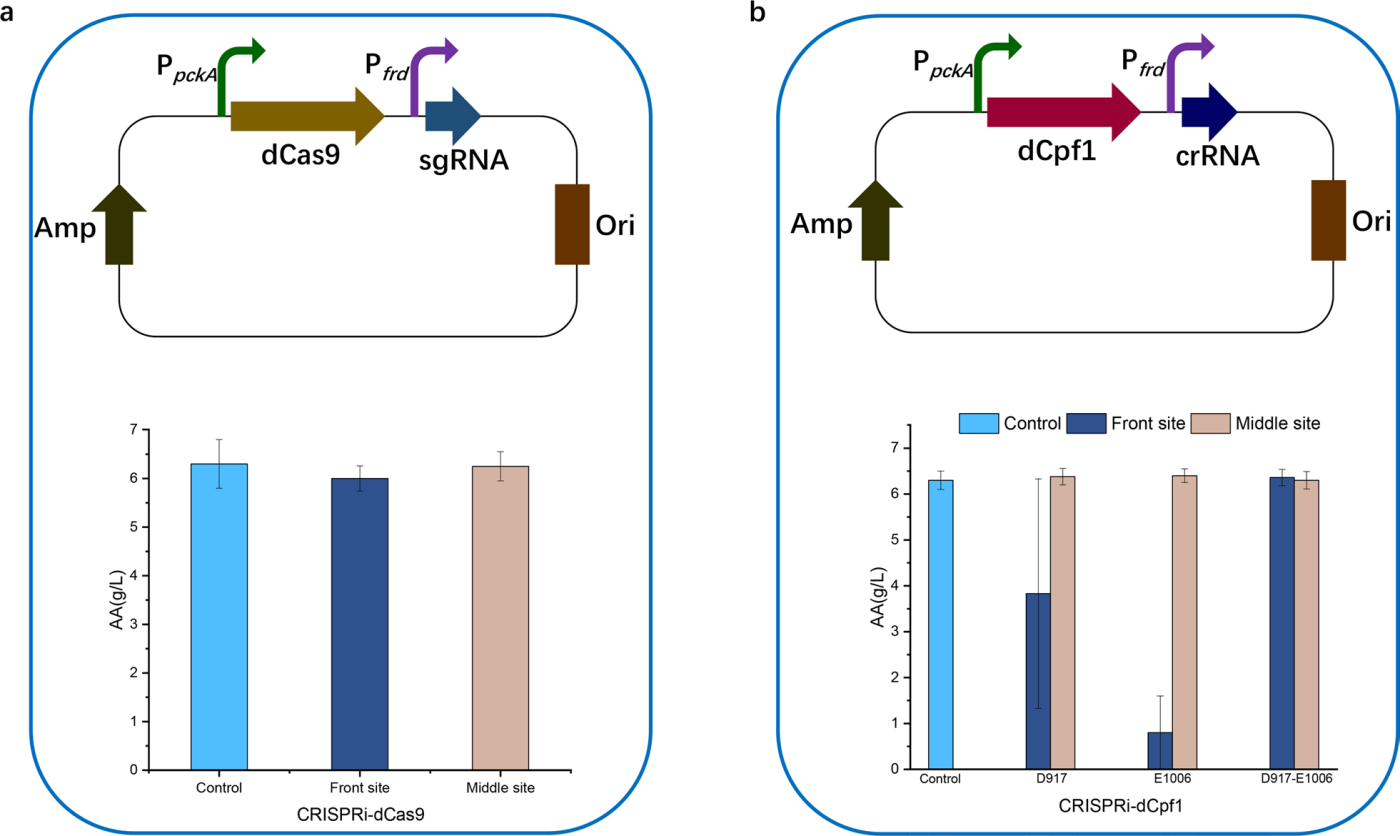
Effects of dCas9 CRISPRi system (**a**) and dCpf1 CRISPRi system (**b**) on acetic acid (AA) production. AA production was measured after fermentation for 48 h. Results are the means of at least three independent assays and error bars indicate the standard deviation

To establish a CRISPRi system based on Cas9, the D10A/H840A mutant of Cas9opt was constructed to disrupt the RuvC and HNH nuclease domains, producing dCas9opt. Since very few *A. succinogenes* promoters have been characterized, there was a limited choice of promoters available to express the sgRNA scaffold, however, since *A. succinogenes* naturally produces abundant SA, it appeared that the *frd* (fumarate reductase) promoter is a high-expression promoter. Therefore, the fumarate reductase promoter was selected to express the gRNA scaffold, to target the acetate kinase (*ackA*) gene (Fig. 3a), encoding acetate kinase, which diverts PEP from SA to acetic acid (AA) production (Fig. 1a). Co-expression of a catalytically-inactivated Cas9, lacking endonuclease activity and a guide RNA, can generate a DNA recognition complex that specifically interferes with transcriptional elongation, RNA polymerase binding, or transcription factor binding (Qi et al. 2013). Two sites in the coding region of the target gene were selected, acting on the front and middle positions of the non-template strand. AA production during fermentation by the resulting mutant was used as a measure of the extent of acetate kinase repression. AA production was not significantly different from the control strain, after mutating the middle position of the target gene and only slightly decreased after mutating the front position (Fig. 3a), indicating that the Cas9-based CRISPRi system had minimal repression activity. Although the Cas9-based CRISPRi system was successfully established, it was ineffective in *A. succinogenes*, possibly because of insufficient expression of Cas9, however, there was no alternative promoter available to test this hypothesis.

### CRISPRi mediated gene repression based on Cpf1 in *A. succinogenes*

The endonuclease activity of Cpf1 can be inactivated by mutation of either D917, or E1006 (Zetsche et al. 2016). To establish a CRISPRi system based on Cpf1, the single and double mutants of Cpf1 were constructed. As for the dCas9-based CRISPRi system, the *frd* promoter expressed the crRNA and the target gene was *ackA* (Fig. 3b). dCpf1 is more effective than Cas9 for targeting the template strand (Mao et al. 2019), so two sites in the coding region of *ackA* were again selected, acting on the front and middle positions of the template strand. The control was a strain with an empty crRNA plasmid. AA production during fermentation was unchanged from control for all mutants at the middle position of *ackA*, indicating no repression effect. Similarly, the double mutant at the front position of *ackA* (D917A-E1006A), had no repression activity, but the single mutations (D917A, E1006A) were effective (Fig. 3b). The E1006A mutation decreased AA production ~ eightfold, whereas D917A only decreased AA production by ~ 1.7-fold. The negligible repressive effect of the double mutant variant may result from the complex structure of the Cpf1 domain; the double mutation may interfere with the RNA processing and/or DNA binding ability of Cpf1, thereby affecting its regulatory activity.

In addition, to confirm the capability of the dCpf1-CRISPRi system for gene repression in *A. succinogenes*, the *lacZ* gene, encoding β-galactosidase, was selected as a target. Light blue mutant colonies were observed on TSB-X-gal plates, suggesting that the CRISPRi-dCpf1 system worked well and could be used for the subsequent experiments (Additional file 1: Fig. S1).

### Gene repression by CRISPR-dCpf1 for increasing SA production

In a previous study, even though a purer SA product was obtained by deleting the *ackA* gene, the SA production decreased, because of stunted growth of *A. succinogenes* (Guarnieri et al. 2017). The *ackA* gene is apparently required for normal cell growth and deletion does not increase SA production (Fig. 1a). However, we hypothesized that *ackA* gene repression may be a potential approach to increase the SA yield, without impairing *A. succinogenes* cell growth. The *ackA* repressor mutant ackA-CRISPRi-1006 decreased AA biosynthesis ~ eightfold, so this strain was used for the subsequent experiments.

**Fig. 4:**
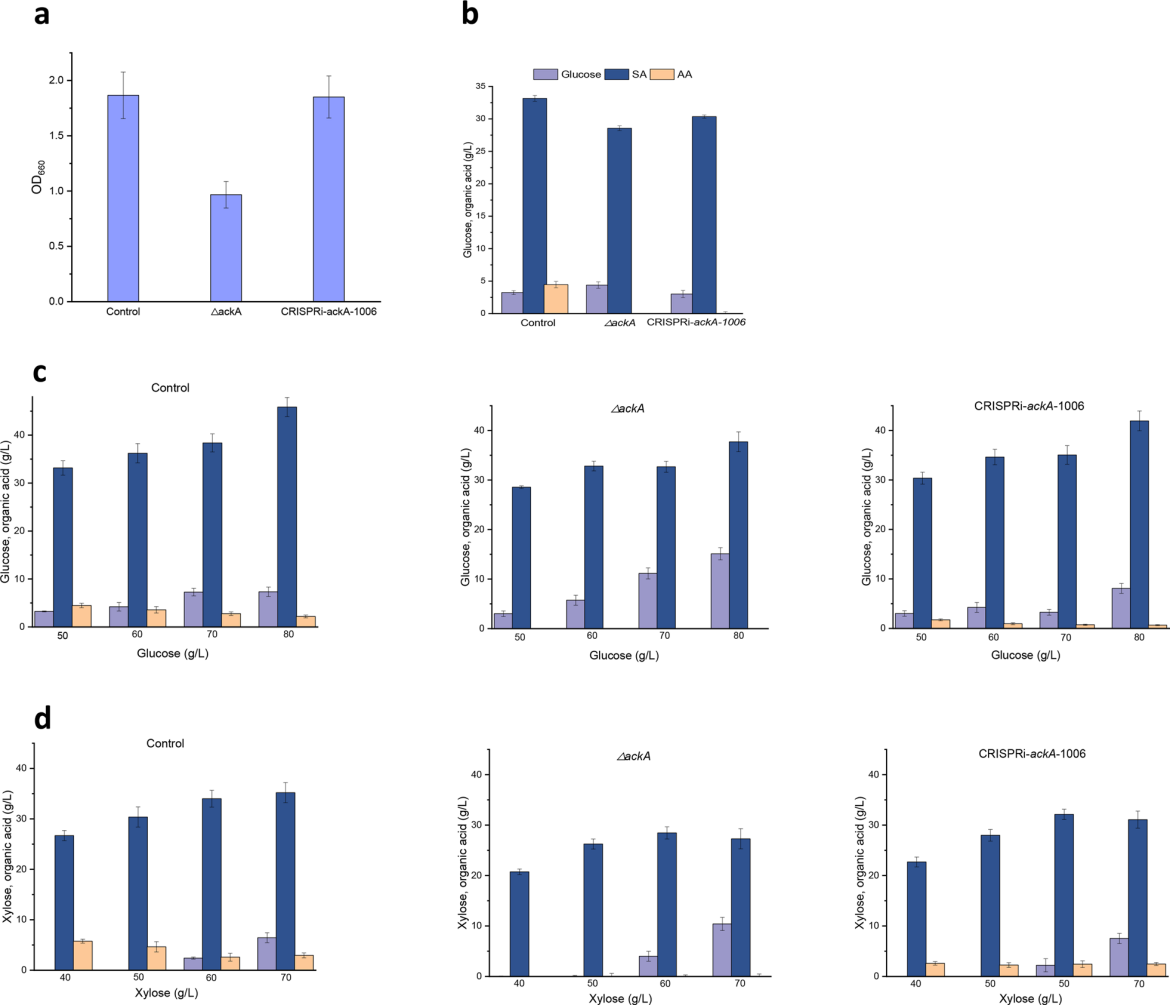
Effects of CRISPRi suppression of the *ackA* gene on *A. succinogenes* strains: cell growth (OD_660_) (**a**); succinic acid (SA) production; residual glucose concentration; acetic acid (AA) production, (**b**); effect of initial medium glucose concentration on SA production (**c**); effect of initial medium xylose concentration on SA production (**d**). AA production was measured after fermentation for 60 h; purple bar, residual glucose concentration; blue bar, SA concentration; orange bar, AA concentration. Results are the means of at least three independent assays and error bars indicate the standard deviation

Cell growth was measured in TSB medium; the growth of the mutant *ackA*-CRISPRi-1006 (OD_660_ 1.851) was similar to that of the control strain (1.866) and higher than that of mutant Δ*ackA* (0.967) (Fig. 4a). This indicated that the CRISPRi system had no effect on cell growth and gene suppression is a potential method to enhance SA production. Organic acid production was then measured by shake-flask fermentation; negligible AA was produced by mutants CRISPRi-*ackA*-1006 and Δ*ackA* and the former produced 6.3% more SA than the latter, although less than the control strain, indicating that down-regulation of *ackA* expression is better than complete knockout (Fig. 4b).

To optimize SA production by the three strains, the effects of different initial glucose concentrations in the fermentation medium (50, 60, 70, or 80 g/L) were determined (Fig. 4c). With increasing glucose concentration, the SA yield increased for all three strains (wild type (WT) from 33.16 to 45.86 g/L, Δ*ackA* from 28.56 to 37.72 g/L and CRISPRi-*ackA*-1006 from 30.36 to 41.93 g/L), i.e., 80 g/L gave the highest SA yield. Negligible AA was produced by Δ*ackA*, suggesting that biosynthesis of AA is almost exclusively catalyzed by acetate kinase. The SA yield of CRISPRi-*ackA*-1006 was higher than that of Δ*ackA,* but not as high as WT, suggesting that the expression level of *ackA* is somehow related to SA biosynthesis. In addition, the AA titer of CRISPRi-*ackA*-1006 was ~ 25% that of WT, demonstrating that down-regulation of *ackA* expression successfully lowered AA production. As the glucose concentration increased, AA production decreased (from 1.74 to 0.66 g/L), possibly because more ATP was produced by the alternative glycolytic pathway, thereby decreasing AA production.

Although bio-based SA production has been successful to some extent, high costs limit its application compared to traditional petrochemical routes. Low cost and abundance lignocellulosic feedstock is attractive as a raw material for producing SA. The hydrolysate of lignocellulosic feedstock mainly contains glucose and xylose, which can be used as carbon sources for microbial fermentation. However, the yield of AA increased with xylose as carbon source for *A. succinogenes* fermentation (Lee et al. 2022), so the effect of different fermentation medium xylose concentrations was determined (40, 50, 60, 70 g/L) (Fig. 4d). With increasing xylose concentration, the SA yield increased (WT from 26.69 to 35.20 g/L, Δ*ackA* from 20.74 to 27.29 g/L and CRISPRi-*ackA*-1006 from 22.69 to 31.08 g/L, a similar trend to that from growth on glucose. The AA yield of WT slightly increased with xylose instead of glucose as carbon source, but decreased with increasing xylose concentration. The AA yield from CRISPRi-*ackA*-1006 did not change with xylose concentration, but was higher than with glucose as carbon source and the SA yields were all lower than with glucose, indicating that *ackA* repression is less effective during growth on xylose than on glucose.

### SA production scale-up by *ackA*-CRISPRi-1006 to a 3 L fermentation

**Fig. 5:**
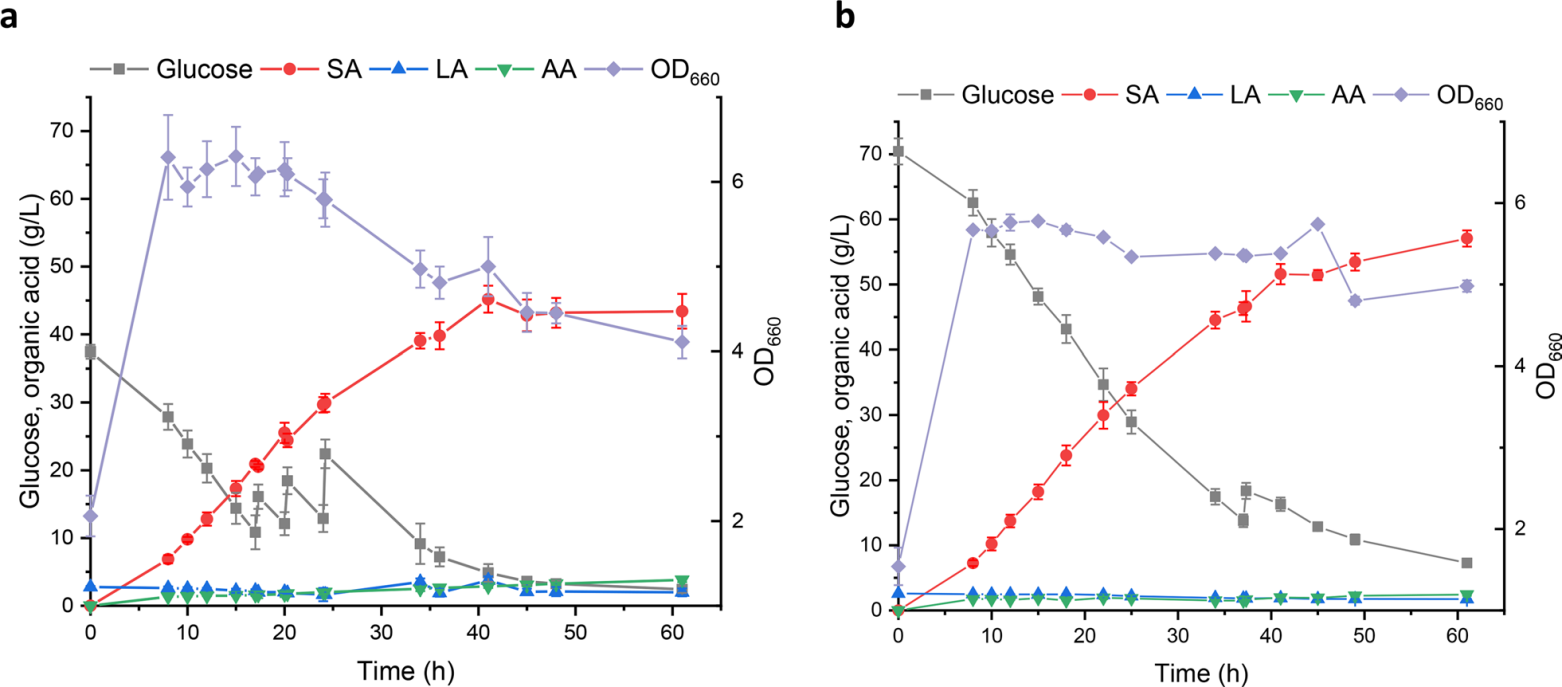
Time courses of SA production in a 3L-scale fed-batch fermentation: 40 g/L initial glucose concentration (**a**); 80 g/L initial glucose concentration (**b**). Results are the means of at least three independent assays and error bars indicate the standard deviation

Time courses of SA production and other parameters were measured in a fed-batch culture with an initial glucose concentration of 40 g/L (Fig. 5a). The maximum OD_660_ and SA titer reached 6.87 and 45.19 g/L, respectively, the SA yield and productivity were 0.71 g/g of SA/glucose and 1.82 g/L/h, respectively and the maximum AA titer was 2.87 g/L. The time course measurements were repeated on a fed-batch culture with an initial glucose concentration of 80 g/L (Fig. 5b). The maximum OD_660_ and SA titer were 5.78 and 57.06 g/L, respectively; the SA concentration with 80 g/L initial glucose was 1.26-fold that from 40 g/L glucose, but it appears that cell growth was inhibited at 80 g/L glucose, possibly because of the higher organic acid concentrations. The SA yield and productivity were 0.79 g/g of SA/glucose and 1.87 g/L/h, respectively and the AA titer (2.44 g/L) was lower, suggesting that down-regulation of AA production is more beneficial for SA production at higher glucose concentrations than the complete removal of AA production and CRISPRi system is a promising method for increasing SA in *A. succinogenes*.

## Discussion

*Actinobacillus succinogenes* is a potential microorganism for industrial SA production, but strain improvement is needed to increase its SA yield to an economically viable level, and this is hampered by limited understanding of the genetic regulatory mechanisms of this species (Long et al. 2021). *Actinobacillus succinogenes* strains have been screened by conventional breeding methods, such as adaptive evolution, mutation breeding and genome shuffling, but these techniques are time-consuming and random, so they cannot change specific genes (Zhang et al. 2020; Hu et al. 2019). Although several gene knockout tools have been established by homologous recombination, this approach is also difficult, complex and time-consuming (Guarnieri et al. 2017; Rhie et al. 2019; Zhang et al. 2019a, b). It is therefore necessary to develop more advanced synthetic biology tools for *A. succinogenes* to obtain improved strains by metabolic engineering. The CRISPR/Cas genome editing system is widely applied to many microorganisms, because of its simplicity and operability. However, double-strand breakage by the endonuclease activity of CRISPR/Cas often decreases cell viability and limits its utility; for primary metabolic genes that are essential for cell growth, gene deletion may further decrease cell viability (Li et al. 2020). However, the CRISPRi system is a programmable gene-knockdown tool that uses an RNA protein complex containing an endonuclease-inactive Cas protein (dCas9/dCpf1) and a single guide to sterically block transcription of the target gene (Banta et al. 2020). In this study, FnCpf1 and codon-optimized Cas9 were expressed successfully in *A. succinogenes*, providing a strong foundation for future application of the CRISPR/Cas genome editing system in *A. succinogenes*. In addition, a CRISPRi system was established for the first time in *A. succinogenes* using CRISPR/dCpf1 without endonuclease activity, achieving strong repression of a specific gene (*ackA*) with no reduction in cell viability. The gene repression vector was obtained easily by replacing a specific crRNA using primers. In addition, the related sgRNA and crRNA elements were expressed successfully in *A. succinogenes* by the *frd* promoter, which appears to be an effective promoter for expression of other exogenous proteins in future research. Overall, the CRISPRi system developed in this study has great potential as a synthetic biology tool for future research on *A. succinogenes*.

Previous metabolic engineering studies of *A. succinogenes* to improve succinic acid production are limited and only knockout of the *pflB* and *ackA* genes has been reported (Guarnieri et al. 2017; Zhang et al. 2019a, b). Acetate kinase (*ackA* gene) catalyzes conversion of acetyl-phosphate and ADP to acetate and ATP, and pyruvate formate lyase (*pflB* gene) catalyzes pyruvate and coenzyme A (CoA) into formic acid and acetyl-CoA in *A. succinogenes* (Fig. 1a). The start of logarithmic cell growth and the onset of SA biosynthesis are delayed by deletion of either gene. For example, deletion of *ackA* decreased the SA titer, yield and productivity, indicating that the removal of heterofermentative pathways either did not enhance carbon flux to SA biosynthesis, or otherwise inhibited it (Guarnieri et al. 2017), in agreement with our results. Pyruvate formate lyase 1-activating protein (*pflA*) can activate *pflB* under anaerobic conditions in *A. succinogenes* (Zhang et al. 2018), but the SA titer (15.78 g/L) was not markedly increased after deletion of *pflA*, and the AA and LA titers increased under aerobic conditions (Zhang et al. 2019a, b). Clearly, simply blocking the C3 pathway does not increase, but rather reduces the SA titer during anaerobic fermentation; it appears that the C3 pathway in *A. succinogenes* is closely involved in cell growth; knockout of *ackA* markedly reduces the biomass of *A. succinogenes*. In this study, suppression of *ackA* expression by CRISPR-dCpf1 completely reversed the cell growth decrease resulting from *ackA* knockout, markedly reduced AA production, and slightly increased SA production. Although SA production was still lower than WT, the superiority of primary metabolic gene suppression over gene deletion suggests that dynamic regulation of competing pathways has clear potential to increase SA production. However, the relationships between SA biosynthesis and competing pathways such as the AA, formic acid and ethanol pathways and the effects of suppressing them remain to be elucidated. The extracellular secretion of acetic and formic acids has an important function in the energy extraction capabilities of *A. succinogenes* (Lexow et al. 2021), further balancing of energy and secreted acid concentrations is a potential way to enhance SA titers in *A. succinogenes*.

## Supplementary Information

**Additional file 1: Figure S1.** Phenotypic characterization of mutant and control strain (The mutant and control strain were streaked on TSB-X-gal plates). **Table S1.** Strains and plasmids used in this study. **Table S2.** The primer sequences used in this study.

## Data Availability

All data generated or analyzed during this study are included in this published article (and its Additional files). All vectors generated in this study can be obtained from the corresponding author on reasonable request.
