# Supplementary material for: Effects of down-regulation of ackA expression by CRISPR-dCpf1 on succinic acid production in Actinobacillus succinogenes
Source: AMB Express. 2023 Jan 26;13:12. doi: 10.1186/s13568-023-01518-x (PMC9880102; doi:10.1186/s13568-023-01518-x)
Supplement: Supplementary file 1 — Additional file 1: Figure S1. Phenotypic characterization of mutant and control strain (The mutant and control strain were streaked on TSB-X-gal plates). Table S1. Strains and plasmids used in this study. Table S2. The primer sequences used in this study. [file 13568_2023_1518_MOESM1_ESM.pdf]

Title: Effects of down-regulation of *ackA* expression by CRISPR-dCpf1 on succinic acid production in *Actinobacillus succinogenes*

Authors: Chunmei Chen, Pu Zheng*

The Key Laboratory of Industrial Biotechnology, Ministry of Education, School of Biotechnology, Jiangnan University, Wuxi 214122, China.

*Corresponding author: Pu Zheng

Tel./ fax: +86 510 8591 8156.

E-mail address: zhengpu@jiangnan.edu.cn.

Additional information

Table Captions

Figure S1 Phenotypic characterization of mutant and control strain (The mutant and control strain were streaked on TSB-X-gal plates)

Table S1 Strains and plasmids used in this study

Table S2 The primer sequences used in this study

Figure S1 Phenotypic characterization of mutant and control strain

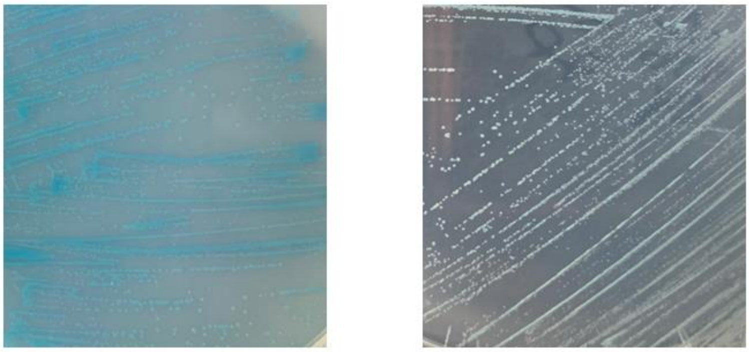

Control *lacZ*-CRISPRi-1006

Table S1 Strains and plasmids used in this study

Strains and plasmids	Description	Reference or source
Strains		
JM109	*E. coli*, cloning strain	Our laboratory
F3-II-F	*A. succinogenes*, wild type	Our laboratory
△*ackA*	*A. succinogenes* derivative, contains *ackA* knockout	This study
CRISPRi-ackA-917	*A. succinogenes* derivative, contains *ackA* repression by dCpf1 of D917A mutation	This study
CRISPRi-ackA-1006	*A. succinogenes* derivative, contains *ackA* repression by dCpf1 of E1006A mutation	This study
Plasmids		
pLGZ922	pLGZ920 derivative	Chen CM et al.2022
pKCcas9dO	contains *cas9* and sgRNA scaffold	Huang H et al. 2015
pDZLcas12a	pDZL803 derivative, contains *FnCpf1*	Zhou YJ et al. 2020
pUCLcrRNA	contains crRNA scaffold	Zhou YJ et al. 2020
pLGZ-cas9opt	pLGZ922 derivative, contains *cas9*opt	This study
pLGZ-pcas9	pLGZ922 derivative, contains *cas9* of vector pCas	This study
pLGZ-kcas9	pLGZ922 derivative, contains *cas9* of vector pKCcas9dO	This study
pLGZ-Cpf1	pLGZ922 derivative, contains *cpf1* of vector pDZL12a	This study
pLGZ-dcas9opt	pLGZ922 derivative, contains *cas9*opt of D10A and H840A mutation	This study
pLGZ-dcas9opt-frd-sgRNA	pLGZ922 derivative, contains d*cas9*opt and sgRNA scaffold under the promoter *frd*	This study
pLGZ-dcpf1-917A-frd-crRNA	pLGZ922 derivative, contains d*cpf1* of D917A mutation and crRNA under the promoter *frd*	This study
pLGZ-dcpf1-1006A-frd-crRNA	pLGZ922 derivative, contains d*cpf1* of E1006A mutation and crRNA under the promoter *frd*	This study
pLGZ-dcpf1-917A-1006A-frd-crRNA	pLGZ922 derivative, contains d*cpf1* of D917A and E1006A mutation and crRNA under the promoter *frd*	This study

Table S2. The primer sequences used in this study

Primer	Sequence (5’ to 3’)	Usage
ackAi-5F	GAGCAATGTTATTTGTTTGACAACCGAC	*ackA* up fragment
ackAi-5R	AGATGAGCTACCGCAGTTAAGAATTAAAAC	*ackA* up fragment
ackAi-3F	GCACAGGATACCGCCCGTCTTTG	*ackA* down fragment
ackAi-3R	GCGTTGGCATAGTTTATTGATCTCGTTATCG	*ackA* down fragment
ackAi-KnF	GTTTTAATTCTTAACTGCGGTAGCTCATCTtcatgaacaataaaactgtctgc	Kanamycin resistant fragment
ackAi-KnR	CAAAGACGGGCGGTATCCTGTGCttagaaaaactcatcgagcatc	Kanamycin resistant fragment
ackAi-outF	GACCACCCGTTTTCTTACGATGG	*ackA* knockout identification
ackAi-outR	GTCGACCGGTACTTCAAGGTTG	*ackA* knockout identification
Cas9opt-plgzhomo-F	*ttatcaatgaggtgatctaga*ATGGATAAAAAATATTCCATC	*Cas9opt* fragment
Cas9opt-plgzhomo-R	*acggccagtgaattcgagctc*TTAATCACCACCTAATTGG	*Cas9opt* fragment
kCas9-plgzhomo-F	*ttatcaatgaggtgatctaga*ATGGACAAGAAGTACTCCA	*kCas9* fragment
kCas9-plgzhomo-R	*acggccagtgaattcgagctc*TCAGTCGCCGCCCAGCTGG	*kCas9* fragment
pCas9-plgzhomo-F	*ttatcaatgaggtgatctaga*ATGGATAAGAAATACTCAATAG	*pCas9* fragment
pCas9-plgzhomo-R	*acggccagtgaattcgagctc*TCAGTCACCTCCTAGCTGA	*pCas9* fragment
dcas9opt-D10A-F	CGGTTTAgcaATCGGTACCAACTCCGTGGG	*Cas9opt* D10A site mutation
dcas9opt-D10A-R	TGGTACCGATtgcTAAACCGATGGAATATTTT	*Cas9opt* D10A site mutation
dcas9opt-H840A-F	TGTGGATgcaATCGTGCCGCAATCCTTCTT	*Cas9opt* H840A site mutation
dcas9opt-H840A-R	GCGGCACGATtgcATCCACATCATAATCGGATA	*Cas9opt* H840A site mutation
Frd-plgzhomo-F	*TGATTAAGAGcgtttaggtttacgtcgctaa*	*frd* promoter fragment
Frd-sgRNA-R	*GAAATTGCAgatatttttctccaataaaaagt*	*frd* promoter fragment
sgRNA-F	*gaaaaatatcTGCAATTTCCATTTGATACGG*	sgRNA scaffold frgment
sgRNA-R	TGAATTCGAGGCACCGACTCGGTGCCACTTTTT	sgRNA scaffold frgment
Plgz-cas9opt-F	GAGTCGGTGCCTCGAATTCACTGGCCGTC	pLGZ920 vector containing cas9opt to linearization

Plgz-cas9opt-R	aacctaaacgCTCTTAATCACCACCTAATTGG	pLGZ920 vector containing cas9opt to linearization
ackAsgRNA1-F	attcgtcgttacggcgcacaGTTTTAGAGCTAGAAATAGCAAG	*ackA* sgRNA1 fragment
ackAsgRNA1-R	tgtgcgccgtaacgacgaatgatatttttctccaataaaaagt	*ackA* sgRNA1 fragment
ackAsgRNA2-F	gttaagaattaaaactaattGTTTTAGAGCTAGAAATAGCAAG	*ackA* sgRNA2 fragment
ackAsgRNA2-R	gttaagaattaaaactaattgatatttttctccaataaaaagt	*ackA* sgRNA2 fragment
12a-plgzhomo-F	*ttatcaatgaggtgatctaga*atgtcgatctaccaagagtt	*cas12a* fragment
12a-plgzhomo-R	*acggccagtgaattcgagctc*tcaattgttgcgattttggacg	*cas12a* fragment
d12a-d917-F	gtcgatcgcacggggggaacggcacctg	*cas12a* D917A site mutation
d12a-d917-R	ccccccgtgcgatcgacaggatgtgcacatcatt	*cas12a* D917A site mutation
d12a-e1006-F	ggtctttgcagacctgaactttggcttcaaa	*cas12a* E1006A site mutation
d12a-e1006-R	agttcaggtctgcaaagaccacaatcgcgttg	*cas12a* E1006A site mutation
crRNA-frdhomo-F	gaaaaatatcAAACGGGTCTAAGAACTTT	crRNA fragment
crRNA-plgzhomo-R	acggccagtgaattcgagctcATAAAACGAAAGGCCCAGTCTTT	crRNA fragment
frd-12ahomo-F	aatcgcaacaattgagagctcCGTTTAGGTTTACGTCGCTAATTTT	*frd* promoter fragment
frd-12ahomo-R	AGACCCGTTTgatatttttctccaataaaa	*frd* promoter fragment
Plgz-12a-30-F	GAgctcgaATTCACTGGCCGTCGTTTTACA	pLGZ920 vector containing cas12a to linearization
Plgz-12a-30-R	GAGCTCtcaattgttgcgattttggacgaa	pLGZ920 vector containing cas12a to linearization
acka-crrna1-F	GGTGTCGCGGAAGATAAAATTAACGGTCTAAGAACTTTAAATAATTTCTACTGTTGTAGATATCGACTGCCA	*ackA* crRNA1 fragment
acka-crrna1-R	CGTTAATTTTATCTTCCGCGACACCATCTACAACAGTAGAAATTATTTAAAGTTCTTAGACCCGTTTgatatttttctcca	*ackA* crRNA1 fragment
acka-crrna2-F	gttttaattcttaactgcggtagGTCTAAGAACTTTAAATAATTTCTACTGTTGTAGATATCGACTGCCAGGCAT	*ackA* crRNA2 fragment

acka-crrna2-R	ctaccgcagttaagaattaaaacATCTACAACAGTAGAAATTATTTAAAGTTCTTAGACCCGTTTgatatttttctcca	*ackA* crRNA2 fragment
LacZ-crRNA-F	cgaccaatcagtcgccggatcaGTCTAAGAACTTTAAATAATTTCTACTGTTGTAGATATCGACTGCCAGGCAT	*LacZ* crRNA fragment
LacZ-crRNA-R	atccggcgactgattggtcgcATCTACAACAGTAGAAATTATTTAAAGTTCTTAGACCCGTTTgatatttttctcca	*LacZ* crRNA fragment
